# Symptoms, mechanisms, and management of long COVID: understanding its prevalence, characteristics, and healthcare challenges in Saudi Arabia

**DOI:** 10.3389/fcimb.2026.1747443

**Published:** 2026-05-12

**Authors:** Abdullah Albelasi

**Affiliations:** Department of Medical Laboratories, College of Applied Medical Sciences, Shaqra University, Shaqra, Saudi Arabia

**Keywords:** immune dysregulaiton, Long COVID, multidisciplinary management, SARS-CoV-2, Saudi Arabia, viral persistance

## Abstract

Long COVID is a prolonged health condition wherein patients continue to experience symptoms long after recovering from infection, so it presents a significant global health challenge with multi-organ involvement. This review provides evidence from several studies to elucidate the prevalence, symptom clusters, and underlying mechanisms of Long COVID. Key findings highlight fatigue (25–73%), cognitive dysfunction, respiratory symptoms (dyspnea: 6.9–47%), cardiovascular symptoms (49.37/1,000), and reproductive symptoms (menstrual irregularities, erectile dysfunction) as predominant manifestations. Mechanistic insights include viral persistence, immune dysregulation, and endothelial dysfunction. The review gives detailed information about prevalence and immunological studies carried out in Saudi Arabia on Long COVID and underscores the importance of longitudinal immune studies, multidisciplinary approaches, biobanks, and global collaborations to improve patient outcomes. Recommendations emphasize tailored therapies, psychological support, and large-scale cohort studies to address heterogeneity and optimize care for Long COVID patients in Saudi Arabia.

## 1 Introduction

The coronavirus disease 2019 (COVID-19) infected about 800 million people worldwide (COVID-19 cases | WHO COVID-19 dashboard). The number of COVID cases in Saudi Arabia is approximately 850,000, and more than 9,000 deaths have been reported. COVID-19 symptoms and health issues that persist for over 4–12 weeks after the initial infection, without a return to baseline health, are called Long COVID (Almalag et al., 2025). The World Health Organization (WHO) formally recognized the condition of lingering effects following an acute severe acute respiratory syndrome coronavirus 2 SARS-CoV-2 infection, defining Long COVID through its Delphi consensus as a “post-COVID-19 condition that occurs in individuals with a history of probable or confirmed SARS-CoV-2 infection, usually 3 months from the onset of COVID-19, with symptoms that last for at least 2 months and cannot be explained by an alternative diagnosis”. Studies reveal that more than 10% of COVID-19 patients experience Long COVID (Taher et al., 2025). Long COVID affects multiple organs of the pulmonary, cardiovascular, renal, endocrine, and gastrointestinal systems, which may result in immunological, neurological, and hematological complications. Further, exhaustion, fatigue, diarrhea, dysfunction of taste and smell, and even depression have been reported (Taher et al., 2025). Even with vaccination and fewer acute infections, the number of Long COVID cases has been increasing. Long COVID in the Middle East, especially in Saudi Arabia, has shown varying prevalence rates and persistent symptoms. This review aims to: (1) describe the range of clinical features and the possible underlying mechanisms of Long COVID based on global evidence; (2) examine the available epidemiological and immunological studies reported from Saudi Arabia; (3) discuss the main challenges related to diagnosis and management within the Saudi healthcare setting; and (4) point out existing gaps in knowledge and suggest areas for future research and improvement in care.

## 2 Methods

### 2.1 Search strategy

The databases -PubMed, Scopus, Web of Science were searched. In addition to these Google Scholar was searched to include additional information. The “Long COVID”, “post-COVID 19 condition”, “SARS-CoV-2”, “Saudi Arabia” using Boolean operation. The search date was from March 2020 to July 2025.

### 2.2 Study selection criteria

The inclusion criteria was peer-reviewed articles on Long COVID studies with Saudi/GCC focus. Editorials, non-English articles and articles with no primary data were excluded.

### 2.3 Data extraction

The extracted data included study characteristics (author, year of publication, and country), study design, sample size, and participant characteristics where available. Information on key outcomes, such as clinical manifestations, prevalence estimates, and reported pathophysiological mechanisms, was also collected. For studies conducted in Saudi Arabia, additional emphasis was placed on extracting epidemiological data, immunological findings, and healthcare-related observations.

### 2.4 Quality assessment

The quality of the included studies was considered during the review process, for study design, sample size, and overall methodology used.

### 2.5 Study selection

**Table:** 

Records identified through database searching: n = 612 Additional records identified through reference lists: n = 38 Total records: n = 650


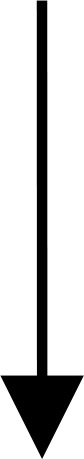


**Table:** 

Records after duplicates removed: n = 520 Records screened (title/abstract): n = 520 Records excluded: n = 355


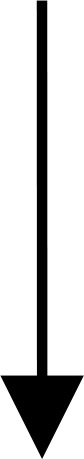


**Table:** 

Full-text articles assessed: n = 165 Full-text articles excluded: n = 68 Irrelevant to Long COVID: 20 Non-English/inaccessible: 8 Duplicate/overlapping data: 10 Not relevant to scope: 30


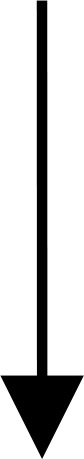


**Table:** 

Primary research studies: n = 55 Total references included in review: n = 79 Non-study references (reviews, guidelines, definitions, tools): n = 24

## 3 The clinical spectrum and pathophysiology of long COVID

### 3.1 Neurological and psychiatric symptoms

Long COVID frequently presents with fatigue and cognitive dysfunction, characterized by impaired memory, attention deficits (“brain fog”), and executive dysfunction. Common neurological symptoms include headaches, anxiety, depression, and sleep disturbance, while rarer manifestations encompass dizziness, stroke, mood disorders, obsessive-compulsive symptoms, paranoia, and PTSD (Spudich and Nath, 2022; Davis et al., 2023). “Brain fog” is a syndrome involving attention deficits, slowed information processing, reduced language fluency, memory lapses, and impaired executive function (Kao and Frankland, 2022). Although mood disorders and anxiety often resolve within around two months, the risks of persistent cognitive impairment, dementia, epilepsy, and psychotic disorders remain elevated for up to two years post-infection (Taquet et al., 2022). The prevalence of brain fog and anxiety has been reported to be significantly higher with the alpha variant of the virus, as compared to the wild-type Wuhan-variant (Spinicci et al., 2022).

The underlying mechanisms involve multiple pathways. SARS-CoV-2 demonstrates neurotropic potential, with viral proteins and RNA detected in brain tissue and neural cell cultures (Stein et al., 2022). Animal studies have further reinforced this hypothesis, with SARS-CoV-2 RNA found in the brains of infected primates, alongside neuroinflammatory changes, hypoxia, and microhemorrhages (Rutkai et al., 2022). Specific viral proteins (ORF6, ORF10) contain neurotoxic sequences that may contribute to neuronal dysfunction (Charnley et al., 2022). Neuroimaging studies reveal structural changes, including reduced gray matter thickness and overall brain volume (Heine et al., 2023), likely mediated by chronic neuroinflammation. Persistent elevation of inflammatory cytokines (e.g., CCL11) and microglial activation may suppress neurogenesis, with animal models showing prolonged neuroinflammation post-infection (Frere et al., 2022). Vascular pathology is evidenced by sustained endothelial dysfunction and abnormal von Willebrand factor levels months after an acute infection (Sánchez-Santillán et al., 2024). These findings suggest that Long COVID’s neurological manifestations result from combined viral neuro-invasion, persistent inflammation, and vascular injury mechanisms.

Neurological symptoms have also been described for Long COVID Saudi Arabia. Patients have reported impaired concentration (“brain fog”), headache, dizziness, and loss of smell or taste (Mahmoud et al., 2021; Alkhotani et al., 2022). The difference between global studies and Saudi studies is that the reports from Saudi Arabia were from cross-sectional studies using questionnaires on self-reported symptoms while several studies reported from other countries have studied potential mechanisms.

### 3.2 Chronic fatigue

Myalgic encephalomyelitis/chronic fatigue syndrome (ME/CFS), is a neuroimmune disorder involving persistent, disabling fatigue; it is notably aggravated by physical or mental activity, a hallmark known as post-exertional malaise. Many patients report flu-like prodromal symptoms that include fever, respiratory/gastrointestinal disturbances, muscle pain, and lymphadenopathy, before ME/CFS onset (Ljungström et al., 2025). Some 58.7% of adult Long COVID patients fulfilled ME/CFS criteria in one study, and 40% of pediatric COVID-19 cases in a cross-sectional analysis developed ME/CFS (Jason and Dorri, 2022). Some of the proposed mechanisms include immune dysregulation and inflammation, as SARS-CoV-2 triggers widespread neuroinflammation, with elevated cytokines (e.g., IL-6, IL-1β) being implicated in both conditions (Yin et al., 2024). Endothelin-1 dysregulation in Long COVID correlates with exertion intolerance, thus mirroring ME/CFS. Retinal microvascular damage and endothelial marker abnormalities (e.g., von Willebrand factor) persist post-infection (Haffke et al., 2022). Metabolic and mitochondrial disruptions observed in Long COVID also contribute to fatigue and cognitive symptoms. Finally, the gut-microbiome-immune axis may contribute to ME/CFS pathophysiology, offering new research directions for Long COVID syndromes (Jurek and Castro-Marrero, 2024). Similar to the brain fog and anxiety, the prevalence of myalgia was higher in the alpha variant than wild-type-Wuhan-variant (Spinicci et al., 2022). Persistent fatigue is also described as a symptom of Long COVID in Saudi Arabian population (Al-Johani et al., 2023) which is in consistent with global studies that have been described above. The Saudi Arabian report used Chalder fatigue scale (CFQ 11) to evaluate the burden of fatigue (Chalder et al., 1993).

### 3.3 Respiratory manifestations of Long COVID

Long COVID patients report experiencing respiratory symptoms, with cough and dyspnea (shortness of breath) being the most common (Iqbal et al., 2025). In one clinical study, 43.4% of patients experienced ongoing dyspnea two months after infection (Carfì et al., 2020). A cohort study found that about 24% of Long COVID patients had dyspnea and dry cough after acute illness (Grewal et al., 2023). These findings emphasize that COVID-19 can lead to long-term respiratory complications that require ongoing clinical attention. Structural lung injury is one of the mechanisms underlying long-term lung damage (Upadhya et al., 2021). Another underlying mechanism is viral persistence and immune dysregulation (Cho et al., 2022). Elevated pro-inflammatory cytokines (IL-1β, IL-6, IL-8) correlate with persistent dyspnea and may contribute to pulmonary fibrosis (Vijayakumar et al., 2022). Proteomic analyses reveal increased apoptosis and epithelial injury-related proteins in Long COVID patients, alongside cytotoxic T-cell activation, suggesting ongoing immune-mediated lung damage (Littlefield et al., 2022).

Respiratory complaints, particularly shortness of breath and reduced exercise tolerance, have been reported in Saudi Long COVID cohorts (AlRadini et al., 2022; Garout et al., 2022; Jabali et al., 2022; Mohammed Salem et al., 2025). All these studies were cross sectional with sample sizes as low as 49 to 314, 821 indicating that these studies provide limited data as compared to studies from other countries that determined pulmonary function with a radiological follow-up.

### 3.4 Cardiovascular system in Long COVID

Cardiovascular complications represent some of the common and enduring Long COVID symptoms, though reported prevalence varies. A multinational study of 56 countries found that 86.04% of Long COVID patients experienced chest pain, palpitations, or fainting (Davis et al., 2023). Analysis of 153,760 COVID-19 survivors showed elevated cardiovascular risks at 12 months, including arrhythmias (49.37/1,000), inflammatory heart disease (2.44/1,000), and ischemic heart disease (18.47/1,000) (Xie et al., 2022). Notably, 30% developed postural orthostatic tachycardia syndrome (POTS) (Fedorowski and Sutton, 2023). Acute-phase cardiac injury may involve troponin elevation (Aikawa et al., 2021) through multiple mechanisms, such as ACE2-mediated viral toxicity causing myocardial damage; microvascular thrombosis (found in 78.6% of autopsies), and cytokine-driven endothelial injury (IL-6, IL-1, TNF-α) promoting clot formation have been proposed (Chen et al., 2020; Pellegrini et al., 2021). Chronic symptoms are reported to stem from persistent viral reservoirs (detected for >4 months) or autoimmunity (anti-ACE2/β2-adrenoceptor/M2 antibodies) (Chen et al., 2020). Though it is believed that cardiovascular effects are associated with the COVID vaccine or variant species differences, studies have shown no association between the cardiovascular events and vaccines or variants (Karimi et al., 2025).

Cardiovascular symptoms such as chest discomfort, palpitations, and exercise intolerance have also been observed in Saudi patients with Long COVID (Alkhotani et al., 2022; Garout et al., 2022; Jabali et al., 2022). These studies were observational that were done using questionnaires without detailed cardiac evaluation.

### 3.5 Gastrointestinal manifestations of Long COVID

Long COVID can lead to persistent gastrointestinal problems, including acid reflux, changes in bowel habit, reduced appetite, and abdominal discomfort (Meringer and Mehandru, 2022). An international survey of patients from 56 countries found that 20.5% experienced chronic diarrhea, while 13.7% reported appetite loss even seven months post-infection (Fernández-de-las-Peñas et al., 2022). In a study of 749 COVID-19 survivors, 29% continued to experience gastrointestinal issues six months after their initial recovery. The most frequently reported problems were “acid reflux/heartburn (16.3%), constipation (11.1%), diarrhea (9.6%), abdominal pain (9.4%), and nausea/vomiting (7.1%)” (Blackett et al., 2022). The potential mechanisms behind GI symptoms are viral persistence in the gut and gut microbiome dysregulation.

Gastrointestinal symptoms have reported in few studies on Long COVID in Saudi Arabia. A study by Al Agran et al. reported that about 28.5% of Long COVID patients presented with GI manifestations like diarrhea and nausea (Al Argan et al., 2022) while a report by Al Maliki et al. showed the prevalence to be around 13.2% (Alzahrani et al., 2025). This variation may be due to underreporting or differences in study focus rather than true variation in prevalence. The first study was a retrospective observational study while the second one was cross section survey conducted using a questionnaire.

### 3.6 Impact of Long COVID on the reproductive system

Research on long COVID’s impact on reproductive health remains limited, though many patients report persistent symptoms. Women commonly experience menstrual irregularities (Phelan et al., 2021; Khan et al., 2022), with some studies suggesting ovarian dysfunction through altered follicular fluid composition (reduced IL-1β and VEGF levels) (Ding et al., 2021). However, other research found no direct ovarian impairment, indicating irregularities may result from systemic inflammation or stress (Herrero et al., 2022). In men, COVID-19 is associated with reduced sperm quality and production, potentially due to viral damage or immune responses (He et al., 2021). A large retrospective study (n=486) found increased risks of ejaculatory dysfunction, decreased libido, and erectile dysfunction (Subramanian et al., 2022). Persistent erectile issues may stem from SARS-CoV-2 remnants in penile tissue disrupting vascular function (Kresch et al., 2021). Notably, ME/CFS patients with Long COVID often exhibit reproductive issues like menstrual abnormalities and PCOS (Thomas et al., 2022).

Evidence on the reproductive effects of Long COVID in Saudi Arabia show that SARS-CoV-2 infection may influence menstrual patterns and hormonal balance in women of reproductive age. Only one cross-sectional study reported changes in menstrual cycle characteristics following COVID-19 infection, including alterations in cycle length, flow, and regularity (Abdel-Moneim et al., 2022). Cultural sensitivities and limited targeted research may explain why there is only one study in Saudi Arabia with regards to the effect of Long COVID on reproductive health.

### 3.7 Musculoskeletal manifestations of Long COVID

Individuals with Long COVID often experience musculoskeletal complications, such as chronic pain, muscle-wasting (sarcopenia), and a reduction in skeletal muscle mass. Research indicates that a substantial number of these patients have muscle-related symptoms (Li et al., 2023). For example, the Linköping COVID-19 Study showed that 28.5% of participants reported limb weakness, and 10.5% experienced generalized muscle weakness (Fernández-de-las-Peñas et al., 2022). Another study revealed that 18.59% of hospitalized survivors developed joint pain, and 15.09% experienced myalgia (muscle pain) six months after infection. Direct viral damage, chronic inflammation with microvascular injury, hypoxia leading to muscle damage, and secondary factors are possible mechanisms for musculoskeletal manifestations. SARS-CoV-2 may directly infect musculoskeletal tissue, as skeletal muscle cells express ACE2 and TMPRSS2, key proteins that the virus uses for entry (Meringer and Mehandru, 2022).

Musculoskeletal complaints, including muscle pain, joint pain, and generalized weakness, are commonly described in Saudi Long COVID patients. It was one of the most prevalent symptom reported by participants in a study reported by Garout et al (Garout et al., 2022). A separate regional study in the Ha’il region also identified muscle pain as a significant contributor to post-COVID discomfort (Almoliky et al., 2025). Similar to other studies on various symptoms, these studies also relied on subjective symptom reporting without incorporating functional assessments or rehabilitation outcomes.

## 4 Studies on Long COVID in Saudi Arabia

### 4.1 Prevalence and clinical studies

Between September and October 2020, a cross-sectional web-based study in Saudi Arabia surveyed 979 individuals who had recovered from COVID-19. Participants, with an average age of 37.7 years and a gender distribution of 53% male and 47% female, responded to a structured Arabic-language questionnaire assessing 20 different symptoms spanning six clinical domains (Khodeir et al., 2021). The questions addressed several areas: general (fatigue, weakness), skin/musculoskeletal (muscle aches, joint pain, rash), neurological/psychological (headache, mood changes, insomnia), special senses (loss of smell/taste, hearing/visual issues), respiratory (cough, dyspnea) and gastrointestinal (nausea, diarrhea, abdominal pain). Symptom severity was measured as “mild/moderate/severe”, and the duration (days post-recovery) was also determined. Fatigue and weakness were observed in 73% of participants with moderate severity, with a mean duration of 7–8 days (n=979). Headache, mood changes, and insomnia were observed in 64%, 41%, and 39%, respectively. Loss of smell was reported by 64% of participants, with 62% reporting it as severe, with a mean duration of 13.9 days. Respiratory signs, such as cough (47%, mild) and dyspnea (43%, moderate), and gastrointestinal symptoms of loss of appetite (42%, moderate) and diarrhea (53%, mild) were also reported by the participants. Participants aged over 40 years were linked to prolonged weakness, insomnia, and loss of smell/taste. The study had a few drawbacks, as it collected self-reported data with no control group, and lacked information about initial illness severity.

Another study on Long Covid in Saudi Arabia used a cross-sectional survey of 744 recovered COVID-19 patients from April 2020–December 2021 (Garout et al., 2022). The participants were recruited two months or more post-diagnosis, with a PCR-confirmed SARS-CoV-2 infection. A questionnaire was used to assess persistent symptoms across different physiological systems (respiratory, cardiovascular, neurological, etc.) using the C19-YRS tool (scored 0–10 for severity). The variables recorded included age, gender, comorbidities, hospitalization status, and disease severity (mild to critical). About 47.5% of participants reported persistent symptoms: >50% had ≥2 symptoms. The most common symptoms were “fatigue (25.4%), headache (15.9%), myalgia (8.5%), anxiety (13.2%) and depression (9.5%), and respiratory issues (dyspnea: 6.9%; cough: 11%)”. The duration of symptoms was more than six months in 47.2% of participants. Similar to the aforementioned study, age was a risk factor with a strong relationship between being more than 50 years of age and comorbidities. However, there was no significant association with gender or disease severity (hospitalization did not correlate). The symptoms were mostly mild to moderate (scores 4–6 on C19-YRS), and neuropsychiatric symptoms (e.g., memory deficits, insomnia) affected 10–15% of the participants. The study collected self-reported data and did not consider vaccination status, and it may have had potential recall bias.

A study on Long COVID by Almalag et al. (2025) used a cross-sectional study design involving 486 COVID-19 survivors in Saudi Arabia. The participants were adults over 18 years of age with PCR-confirmed COVID-19, recruited from November–December 2020 via online questionnaires distributed through social media. Demographics (age, sex, smoking status, etc.), comorbidities (e.g., hypertension, diabetes), infection details such as severity (mild to life-threatening), hospitalization, recovery duration, and long COVID symptoms, such as fatigue, breathlessness, anosmia, and muscle/joint pain, were recorded. Of the study participants, 61% reported persistent symptoms after recovering from COVID-19. The most common of these were “fatigue (56%), breathlessness (47%), loss of smell (44%), and muscle aches (40%). The study population was 71% female, and 43% were between 18 and 30 years old.” Comorbidities, such as hypertension, diabetes, and asthma, were present in 30% of participants. While those with comorbidities were generally among older participants (51–60 years), the study found no significant link between these pre-existing conditions and most Long COVID symptoms, with the exceptions of chest pain and tachycardia. Recovery duration was 37% and it took >1 year for the participants to recover; recovery was not affected by comorbidity status. Study limitations were potential bias from online self-reporting and vaccination status not being considered.

A separate cross-sectional investigation carried out at King Abdulaziz Medical City in Jeddah between September and December 2021 also identified comparable patterns in the persistence of COVID-19-related symptoms (Jabali et al., 2022). This study recruited 327 adults (≥18 years) with PCR-confirmed COVID-19, via phone interviews at least six months post-infection. Data on demographics (age, gender, BMI, smoking status, physical activity), comorbidities (diabetes, hypertension, respiratory diseases, etc.), persistence symptoms post-recovery (e.g., anosmia, fatigue), and treatment details (hospitalization, ICU admission, oxygen use, vaccination status) were collected. The study results show that 49.1% of participants reported persistent symptoms. Among those frequently reported symptoms, anosmia accounted for 22.6%, and ageusia for 19.2%, followed by cough (11.6%), fatigue (9.1%), and alopecia (7.3%). Unlike other studies, gender has a significant influence on persistent long COVID symptoms, with females reporting higher rates of anosmia, ageusia, and hair loss. The study also reported that hospitalization and ICU admission were linked to persistent symptoms, and 20.8% of participants required oxygen, significantly associated with persistent symptoms. The study did not determine the impact of vaccination on persistent symptoms.

Finally, a comparative cross-sectional study conducted in Hail, Saudi Arabia from May–July 2023 compared the short-term (infected ≤1 year) and long-term (infected >1 year ago) impact of COVID on 292 adults categorized into different groups (Althomali et al., 2024). A medical outcomes study “short form 36 (SF-36)” assessed health-related quality of life (HRQoL) across eight domains (e.g., physical functioning, social functioning). The “HAM-A” (Hamilton Anxiety Scale) was used to measure psychological outcomes. The short-term (≤1 years post-infection) and long-term impact (>1 years post-infection) were significantly lower on social functioning, with no significant psychological outcomes.

A summary of all the studies is given in **Table 1**. As seen in the table, the prevalence rates of symptoms vary among different reports. Hence, a detailed meta-analysis may provide more insight to identify the causes for the wide variation in prevalence.

**Table 1:** Prevalence of different symptoms of Long COVID in Saudi Arabia.

Symptom	Prevalence	Study (Reference)
Fatigue	73% (moderate severity)	(Khodeir et al., 2021)
	25.4%	(Garout et al., 2022)
	56%	(Almalag et al., 2025)
	9.1%	(Jabali et al., 2022)
Weakness	73% (moderate severity)	(Khodeir et al., 2021)
Headache	64%	(Khodeir et al., 2021)
	15.9%	(Garout et al., 2022)
Mood changes	41%	(Khodeir et al., 2021)
Insomnia	39%	(Khodeir et al., 2021)
	10-15%	(Garout et al., 2022)
Loss of smell (anosmia)	64% (62% severe)	(Khodeir et al., 2021)
	44%	(Almalag et al., 2025)
	22.6%	(Jabali et al., 2022)
Loss of taste (ageusia)	19.2%	(Jabali et al., 2022)
Muscle aches (myalgia)	8.5%	(Garout et al., 2022)
	40%	(Almalag et al., 2025)
Joint pain	Reported in categories	(Khodeir et al., 2021)
Respiratory issues (cough)	47% (mild)	(Khodeir et al., 2021)
	11%	(Garout et al., 2022)
	11.6%	(Jabali et al., 2022)
Dyspnea (breathlessness)	43% (moderate)	(Khodeir et al., 2021)
	6.9%	(Garout et al., 2022)
	47%	(Almalag et al., 2025)
Gastrointestinal (diarrhea)	53% (mild)	(Khodeir et al., 2021)
Loss of appetite	42% (moderate)	(Khodeir et al., 2021)
Anxiety	13.2%	(Garout et al., 2022)
Depression	9.5%	(Garout et al., 2022)
Hair loss	7.3%	(Jabali et al., 2022)
Chest pain	Reported as linked to comorbidities	(Almalag et al., 2025)
Tachycardia	Reported as linked to comorbidities	(Almalag et al., 2025)

### 4.2 Immunological studies in Saudi Arabia

There have been no long-term immunological studies on COVID-19 in Saudi Arabia, but a few short-term studies related to immunological changes in COVID have been carried out in the country. One notable study with a short follow-up period examined humoral antibody responses in hospitalized COVID-19 patients (with varying severity), and vaccinated volunteers from two tertiary hospitals in Saudi Arabia. It found that vaccinated subjects exhibited significantly higher IgG levels against the SARS-CoV-2 spike protein compared to naturally infected patients, regardless of disease severity (Alshahrani et al., 2025). Another study investigated the antibody immune responses induced by the BNT162b2 (Pfizer-BioNTech) and ChAdOx1 (Oxford/AstraZeneca) vaccines in Riyadh. It reported that anti-SARS-CoV-2 spike IgG antibodies were detected in most subjects after the prime dose, peaked after a booster, and remained elevated for six months. Although antibody levels decreased after one year, they increased significantly following a third dose. The study also noted higher IgG titers in previously infected individuals after the initial dose and at six months, particularly among younger subjects (Alharbi et al., 2022). A cross-sectional study conducted in Sakaka City assessed the seroprevalence of anti-SARS-CoV-2 IgM and IgG among both symptomatic and asymptomatic individuals, as well as vaccinated and unvaccinated people. It found statistically significant correlations between vaccination status—especially after a second dose—and IgM/IgG positivity (Taha et al., 2022). A study released in July 2025 examined how healthy individuals in Saudi Arabia responded to a third dose of an mRNA-based COVID-19 vaccine at the biochemical and hematological levels. The findings revealed the enduring elevation of IgG and IgA antibodies over a six-month period, indicating a swift and durable immune activation following booster administration (Bawazir et al., 2025). Another study involving healthcare workers compared the efficacy of different vaccines, finding that both the Pfizer (BNT162b2) and AstraZeneca (ChAdOx1) vaccines showed declining neutralizing antibodies after six months. However, T-cell responses persisted (Mubarak et al., 2022).

These studies provide a comprehensive overview of the research conducted in Saudi Arabia regarding SARS-CoV-2 antibody responses in both infected and vaccinated populations, addressing aspects such as antibody persistence, comparative responses between natural infection and vaccination, and the impact of booster doses. A summary of immunological studies in Saudi Arabia is summarized in **Table 2**.

**Table 2:** Studies on antibody/immune responses in infected and vaccinated subjects in Saudi Arabia.

Key findings	Drawbacks	Study references
- Vaccinated subjects had higher IgG levels against SARS-CoV-2 spike protein than naturally infected patients, regardless of disease severity.	- Short follow-up period. - Focused only on hospitalized patients and vaccinated volunteers.	(Alshahrani et al., 2025)
- Anti-SARS-CoV-2 spike IgG peaked after a booster and remained elevated for 6 months. - Higher IgG titers in previously infected individuals, especially younger subjects.	- Limited to two vaccine types (BNT162b2 and ChAdOx1). - No long-term (>1 year) data.	(Alharbi et al., 2022)
- Significant correlation between vaccination (especially after a second dose) and IgM/IgG seropositivity in symptomatic/asymptomatic individuals.	- Cross-sectional design. - Did not track symptom persistence or clinical outcomes.	(Taha et al., 2022)
- Sustained increases in IgG and IgA levels for 6 months after a third mRNA vaccine dose.	- Small sample size. - Limited to healthy participants (no immunocompromised or elderly groups).	(Bawazir et al., 2025)
- Declining neutralizing antibodies after 6 months, but persistent T-cell responses with Pfizer and AstraZeneca vaccines.	- Limited to healthcare workers. - No variant-specific analysis.	(Mubarak et al., 2022)

### 4.3 Clinical trials on Long COVID in Saudi Arabia

A Phase 2 multicenter, open-label study (NCT04915300) is currently investigating the safety and efficacy of oral apabetalone in patients with Long COVID, focusing on functional capacity (6-minute walk test) and symptoms such as fatigue and cognitive impairment, with Saudi Arabia serving as a key recruitment site (Study Details | NCT04915300 | Apabetalone for Pulmonary Arterial Hypertension | ClinicalTrials.gov). As of now, no completed results from this trial have been published, and additional details on ongoing or incomplete clinical trials are provided in the **Supplementary Data sheet 1**.

## 5 Discussion

The diagnosis and management of Long COVID face significant challenges due to the variety of symptoms, unclear pathophysiology, and the unavailability of standardized biomarkers (Abbas et al., 2025). Presently, diagnosis is based on one’s clinical history due to the lack of biomarkers (Abbas et al., 2025). Potential biomarkers such as micro-clots, immune dysregulation, and viral persistence are yet to be explored (Lai et al., 2023). Given the similar and overlapping symptoms, it is important to be cautious when differentiating Long COVID from conditions like “myalgic encephalomyelitis/chronic fatigue syndrome (ME/CFS), dysautonomia (such as postural orthostatic tachycardia syndrome, or POTS), and mast cell activation syndrome” (Wong and Weitzer, 2021). A thorough understanding of pathophysiology related to viral persistence, autoimmune disorders, damage to the endothelium, and mitochondrial dysfunction is needed (Gupta et al., 2025). It is important to identify reliable biomarkers, such as inflammatory markers, autoantibodies, and micro-clotting, for diagnosis and subtyping. Additionally, clarification is needed of the mechanisms underlying cognitive dysfunction (often referred to as “brain fog”), dysautonomia (e.g., postural orthostatic tachycardia syndrome or POTS), and neuroinflammation (Martins et al., 2025). Validated immune panels for diagnosing long COVID must include cytokines and autoantibodies. Validation of tests and standardization are needed before widespread clinical use.

There is a relative lack of large-scale, long-term longitudinal immune studies on COVID-19 in Saudi Arabia compared to global research efforts. Although some studies have been conducted, significant gaps remain in understanding the durability of immunity, variant-specific responses, and population-wide immune trends over time. Saudi Arabia has prioritized diagnostics, vaccination campaigns, and outbreak control over long-term immune monitoring. Most early studies were cross-sectional, focusing on single-time-point antibody surveys. Unlike the UK, which has the COVID Symptom Study (called the Zoe Health Study and now closed), or the US with NIH-funded cohorts (Home | RePORT), Saudi Arabia lacks dedicated large cohorts and a national longitudinal cohort that tracks immunity over several years. Most studies in the country have short follow-up periods of 6–12 months (Alharbi et al., 2022; Mubarak et al., 2022; Taha et al., 2022; Alshahrani et al., 2025; Bawazir et al., 2025).

Additionally, Saudi Arabia has a large expatriate population, making long-term follow-up challenging due to mobility. Many studies tend to focus on healthcare workers rather than the general population. There are few studies that track antibody and T-cell decay beyond one year, and there is limited data on natural infections combined with repeated booster doses. Most neutralization studies focus on early variants such as Delta and Omicron BA.1, rather than newer sub-lineages. Furthermore, there is a scarcity of studies on immunocompromised or elderly populations in Saudi Arabia.

Addressing Long COVID’s complexity requires a multidisciplinary approach. Establishing biobanks with high-quality specimens (serum, tissues, PBMCs) enables multi-omics analysis of viral persistence, autoantibodies, and immune dysregulation, while standardizing longitudinal sample collection protocols (Abdelhafiz et al., 2022). Large-scale cohort studies tracking diverse populations should incorporate prospective designs, deep phenotyping of symptom clusters/organ dysfunction, and appropriate control groups to identify risk factors like autoimmunity and viral load (Saleh and Adly, 2023). Advanced immuno-profiling using single-cell multi-omics, high-dimensional flow cytometry, and systems immunology can decode immune signatures guiding personalized therapies (Stephenson et al., 2021). Projects like COVIDome (Sullivan et al., 2021) and Stanford IMPACC (Ozonoff et al., 2024) demonstrate this potential, though challenges remain such as symptom heterogeneity, limited animal models, and insufficient diverse population studies (Alshahrani et al., 2025).

The integration of virology and immunology along with long COVID clinics are crucial for diagnosis and treatment. Overlapping symptoms make diagnoses challenging. Virology labs detect persistent viral particles and the reactivation of other viruses (Kostaki, 2025). Immunology labs can identify dysregulated immune response such as autoantibodies, cytokine profiles and T cell abnormalities for the personalized treatment of different subtype patients (Lázár-Molnár and Peterson, 2025). As research advances, the importance of future directions and clinical implications are crucial to enhance diagnoses, treatment, and patient care. Advances in pathophysiology, targeted therapies, and care models will be critical to reducing the burden of this complex condition. A clear understanding of pathophysiology is essential, including mechanistic studies of viral persistence, immune dysregulation, autoimmunity, endothelial dysfunction, and alterations in the microbiome. Further, challenges in the healthcare system include resource allocation and specialized Long COVID clinic and laboratory establishments (Adam et al., 2025). Global collaboration and data-sharing initiatives in Long COVID conditions from all over the world through WHO Long COVID case reports are important and can help us make progress in understanding this syndrome (Cheng et al., 2025). The integration of virology and immunology along with long COVID clinics help to monitor high-risk patients proactively and the response to treatment can be monitored to mitigate Long COVID risks. It will help to unravel the complexities of Long COVID, offer effective treatment, and ensure better care for patients.

## 6 Conclusion

The evidence presented in this review confirms that Long COVID is a significant and complex public health challenge in Saudi Arabia, mirroring global trends while presenting unique regional characteristics. The analysis of different studies shows that a substantial portion of the Saudi population continues to struggle with persistent symptoms long after the acute phase of infection has passed. Fatigue remains the most dominant and debilitating complaint, often accompanied by “brain fog,” respiratory distress, and musculoskeletal pain.

While research from other countries reports the biological drivers of these symptoms by identifying viral persistence, chronic immune dysregulation, and vascular injury as primary mechanisms, the research in the Kingdom is still largely in its descriptive phase. Most current Saudi studies rely on cross-sectional, self-reported data, which, while valuable for identifying prevalence, provide limited insight into the long-term biological changes occurring in these patients. Hence, a transition from merely cataloging symptoms to conducting rigorous, longitudinal research is recommended. There is an urgent need for dedicated biobanks and large-scale cohorts that track the immune responses of Saudi patients over years rather than months. Furthermore, the management of Long COVID cannot remain fragmented. Patients require a multidisciplinary approach that integrates pulmonary, cardiac, and neurological care with robust psychological support to address the diverse multi-organ impact of the condition. Ultimately, bridging the gap between clinical observation and laboratory investigation will be essential. By investing in structured national registries and contributing to global clinical trials, Saudi Arabia can not only improve outcomes for its own citizens but also become a vital contributor to the worldwide effort to understand and treat this enduring post-viral syndrome.
