# Supplementary material for: Symptoms, mechanisms, and management of long COVID: understanding its prevalence, characteristics, and healthcare challenges in Saudi Arabia
Source: Front Cell Infect Microbiol. 2026 May 12;16:1747443. doi: 10.3389/fcimb.2026.1747443 (PMC13201512; doi:10.3389/fcimb.2026.1747443)
Supplement: Supplementary file 1 [file DataSheet1.pdf]

Ongoing/incomplete clinical trial on Long COVID in Saudi Arabia

There is an incomplete clinical trial with ID NCT05799534, titled "Post-Coronavirus Disease of 2019 (COVID-19) Rehabilitation Clinics in Saudi Arabia." It was a project sponsored by Majmaah University. It was designed as a single-group assignment, open-label interventional study. The study aimed to evaluate the effectiveness of a comprehensive physical therapy program in improving both the physical capabilities and the psychoemotional state of Long COVID-19 patients. A four-week structured rehabilitation program was designed for the participants to attend three times a week for 45 to 60 minutes per session. The program consisted of essential therapies, including breathing exercises, muscle strengthening, aerobic workouts, and mobility/gait training. However, the trial had not yet begun recruiting participants. Its last verified status on April 2023, shows that the study was "Not yet recruiting." The trial was to be completed by July 2023. Hence, the trial did not proceed as scheduled, and consequently, no results have been reported suggesting that study has been stopped (Study Details | NCT05799534 | Post-Coronavirus Disease of 2019 (COVID-19) Rehabilitation Clinics in Saudi Arabia | ClinicalTrials.gov).

Another ongoing trial is “A Phase 2 Study of Apabetalone in Subjects With Long -COVID" (NCT04915300), which is an international effort specifically focused on finding a treatment for Long COVID, with Saudi Arabia playing a crucial role as a primary investigative location (Study Details | NCT04915300 | Apabetalone for Pulmonary Arterial Hypertension | ClinicalTrials.gov). The study is designed as a Phase 2, multi-center, open-label trial to assess the safety and efficacy of the investigational drug apabetalone in patients suffering from Long COVID-19 conditions. The drug is administered orally once a day. Saudi Arabia is vital to this research as the study is being conducted at multiple sites across the Kingdom. The primary goal is to see if apabetalone can significantly improve patients' functional capacity, measured by the distance they can walk in six minutes (6-Minute Walk Test), after 12 weeks of treatment. Secondary objectives are aimed at evaluating the drug's impact on persistent and debilitating Long COVID symptoms, such as fatigue, pain, and cognitive function (brain fog), which severely affect a patient's quality of life. The inclusion of Saudi Arabian centers highlights the Kingdom's commitment to contributing to global research on this chronic post-viral condition. The study is currently recruiting .

References

Study Details | NCT04915300 | Apabetalone for Pulmonary Arterial Hypertension | ClinicalTrials.gov Available at: https://clinicaltrials.gov/study/NCT04915300 [Accessed March 25, 2026].

Study Details | NCT05799534 | Post-Coronavirus Disease of 2019 (COVID-19) Rehabilitation Clinics in Saudi Arabia | ClinicalTrials.gov Available at: https://clinicaltrials.gov/study/NCT05799534 [Accessed October 15, 2025].
